# lncRNA TSPEAR-AS2, a Novel Prognostic Biomarker, Promotes Oral Squamous Cell Carcinoma Progression by Upregulating PPM1A via Sponging miR-487a-3p

**DOI:** 10.1155/2021/2217663

**Published:** 2021-07-17

**Authors:** Yi-chao Xia, Jun Cao, Jing Yang, Ying Zhang, Yong-sheng Li

**Affiliations:** Department of Oral and Maxillofacial Surgery, The First People's Hospital of Yunnan Province, The Affiliated Hospital of Kunming University of Science and Technology, Kunming, Yunnan, China

## Abstract

**Background:**

Long noncoding RNA (lncRNA) critically impacts the modulation of tumor developments and progressions. Our study is aimed at investigating the expressing patterns, clinical significance, and biological roles of lncRNA TSPEAR-AS2 (TSPEAR-AS2) in oral squamous cell carcinoma (OSCC). *Material and Approach*. The expressing states achieved by TSPEAR-AS2 were examined in OSCC specimens and cell lines by RT-PCR. The clinical significance of TSPEAR-AS2 was statistically analyzed. OSCC proliferating, invading, and migrating processes were examined with the use of wound healing assays, transwell, colony formation, and cell counting kit-8. Additionally, the downstream molecular mechanism of TSPEAR-AS2 in OSCC was explored.

**Results:**

TSPEAR-AS2 was overexpressed in OSCC tumors and cells. High TSPEAR-AS2 was associated with advanced TNM stage. Patients with high TSPEAR-AS2 expression displayed a shorter disease-free survival and total survival of OSCC patients than those with low TSPEAR-AS2 expressing level. It was found that knockdown of TSPEAR-AS2 could inhibit the proliferating, invading, and migrating processes pertaining to OSCC cells. Luciferase reporter tests and RNA pull-down results revealed that TSPEAR-AS2 enhanced the expressions of PPM1A by regulating miR-487a-3p, and TSPEAR-AS2 could be adopted as a miR-487a-3p sponge to inhibit PPM1A expression.

**Conclusion:**

Our study highlighted the significance of the TSPEAR-AS2/miR-487a-3p/PPM1A axis within OSCC progression and offered a novel biomarker and novel strategies for OSCC treatments.

## 1. Introduction

Oral squamous cell carcinoma (OSCC), ranking among the top eight causes of cancer-related death globally, takes up >90% of head and neck cancer, affecting more than 400 000 people every year [1, 2]. The two main risk factors are alcohol and smoking for OSCC. Poor oral hygiene is also found to be involved in the aetiology of OSCC [3]. Despite the fact that great advances have been made in surgical techniques and chemoradiation therapy, the 5-year survival rate for OSCC patients remains very low [4, 5]. The inability for diagnosing in the preliminary phase refers to the main reason for the unfavorable outcome of OSCC cases [6, 7]. It has been confirmed the involvements of multiple genetic and epigenetic abnormality in OSCC progressions, but the molecular mechanisms involved in OSCC tumorigenesis remain largely unclear.

Long noncoding RNAs (lncRNAs), with the transcription based on RNA polymerase II, are defined as transcripts containing >200 nucleotides [8]. More and more evidences have demonstrated that lncRNAs as one type of transcription factor display vital roles in diverse biological procedures [9, 10]. The dysregulation of lncRNAs is also demonstrated with the involvement inside the occurrence and progression of several tumors [11, 12]. For instance, overexpression of lncRNA PART1 promoted the proliferating and metastasis processes of lung carcinoma cells by sponging miR-17-5p [13]. lncRNA LINC00844, a highly expressed lncRNA in hepatocellular carcinoma, was shown to be a tumor inhibitor within hepatocellular carcinoma and suppress the metastasis of tumor cells via targeting AZGP1 [14]. In OSCC, lncRNA LINC01929 was demonstrated to strengthen the ability of OSCC cells in the proliferation and metastasis via targeting the miRNA-137-3p/FOXC1 axis [15]. Although a lot of human lncRNAs have been reported to be abnormally expressed in OSCC so far, the physiological functions of most lncRNAs have remained largely unclear.

Previously, lncRNA TSPEAR-AS2 was shown to display regulatory effects on hypoxia-induced pulmonary artery hypertension in cellular levels [16]. Recently, Ma and his group firstly reported TSPEAR-AS2 as a gastric cancer-related lncRNA which served as an oncogene and suppressed the metastasis of tumor cells via suppressing GJA1 expression [17]. However, the potential functions of TSPEAR-AS2 in other tumors have not been investigated. In this study, we provided evidences that TSPEAR-AS2 was an overexpressed lncRNA in OSCC specimens and may be a novel biomarker for OSCC patients. In addition, we also studied the function of TSPEAR-AS2 in OSCC as well as its potential mechanisms.

## 2. Materials and Methods

### 2.1. Collection of Human Clinical Specimens

The researchers carried out the collection of ninety-five clinically-related OSCC tumor tissue and paired nearby nontumor tissue in the First People's Hospital of Yunnan Province. The tissue received the collection in the surgical process, and then it underwent the storage within liquid nitrogen or under the temperature of -80°C to be employed subsequently. All the patients verified as OSCC according to histopathological evaluation were operated on at the First People's Hospital of Yunnan Province. This study was performed following a protocol approved by the Ethics Committee of The First People's Hospital of Yunnan Province. The respective patient provided written informed consent for participation.

### 2.2. Cell Culture and Transfection

The Cell Bank of the Chinese Academy of Sciences (Shanghai, China) offered six OSCC cell lines (SNU1041, FADU, HSU3, SCC25, SCC9, and SCC4) and the NHOK cell lines. All cells received the culture within DMEM (Invitrogen, Shenzhen, Guangdong, China) with 10% FBS (Biocyto, Guangzhou, Guangdong, China) under the temperature of 37°C in 5% CO_2_ and saturated humidity. Scrambled shRNA of TSPEAR-AS2 (sh-NC), TSPEAR-AS2 shRNAs (sh-TSPEAR-AS2-1 and sh-TSPEAR-AS2-2), miR-487a-3p mimic (NC mimics), and miR-487a-3p inhibitors (NC inhibitors) was purchased from GenePharma (China). Next, the mentioned received the transfection to SCC25 and FADU cell with the use of Lipofectamine 2000 (Invitrogen/Thermo Fisher Scientific).

### 2.3. RNA Extraction and RT-qPCR

The researchers adopted TRIzol reagent for obtaining overall RNA from specimens and cells. By the use of the PrimeScript RT reagent kit, 3 *μ*g RNA overall received the reverse transcription to cDNA. RT-qPCR was performed with the use of FastStart Universal SYBR Green Master (Roche, Pudong, Shanghai, China) on a Bio-Rad RT-PCR cycler (Roche, Pudong, Shanghai, China). GAPDH and U6 became the control to normalize mRNA's and miRNA's expressing levels, separately. Relative expression values of genes were analyzed by the 2^−*ΔΔ*Ct^ approach.  Table 1 lists the primer sequence of RNAs.

### 2.4. Cell Counting Kit-8 (CCK-8) Assay

To analyze the growth of the OSCC cell lines, the CCK-8 test (Dojindo Laboratories) was performed by complying with the producer's protocol. OSCC cells were cultured in 96-well plates overnight. After 1 d, 2 d, 3 d, and 4 d, cells were added with CCK-8 (Lifusai, Nanjing, Jiangsu, China). Optical density at 490 nm was measured using a microplate reader, and data were expressed as absorbance values. GraphPad Prism 8 software (GraphPad Software, Inc.) was used to plot the cell growth curve. The tests were carried out no less than 3 times.

### 2.5. Colony Formation Assays

0.5 × 10^3^ cells received the inoculation to a 12-well plate and then the 10-day culture. Fresh medium on the 5th day was used to replace the original medium. After the incubating process, the researchers adopted PBS for rinsing cells. Next, the 5 min immobilizing process for cells was carried out by using 4% paraformaldehyde, and then the 30 s staining process by using 0.1% crystal violet was used. Finally, the counting and recording processes were used for the number of colonies (over 50 cells).

### 2.6. EdU Incorporation Assays

Cells received the culture within 24-well plates, and the researchers introduced 10 *μ*M EdU to each well. Subsequently, 4% formaldehyde was used to fix the cells for 30 min. 48 h later, 50 *μ*M of EdU labeling medium was introduced to the respective well, and the cell received the 8 h incubation. Next, the cells were stained with Hoechst 33342 for 20 min and were captured. Eventually, under the microscope, EdU-positive cells received the observation and the counting process.

### 2.7. Wound Healing Assay

The researchers carried out the seeding and culturing process for the cell within a confluent monolayer in a rectangular cell culture plate. The cell received the culture till the confluence reaching nearly 100%. 10 L pipette tips were used to create cell wounds. Moreover, in FBS-free F12K medium (Procell, Nanjing, Jiangsu, China), cells were then cultivated. At 24 h after culture, an inverted microscope was used to measure the width of the wounds. Gap distance was quantified using NIH ImageJ software version 1.50.

### 2.8. Transwell Assay

Using transwell chambers, the ability of cell invasion was examined. A total of 2 × 10^4^ cells was supplemented to the upper compartment of each transwell chamber (pore size: 8 *μ*M; Corning, Haidian, Beijing, China), and 600 *μ*L of medium containing 10% FBS received the addition into the compartment which was relatively low. When 24 h incubating process was achieved under room temperature, the researchers employed a cotton swab for scraping cells on the upper chamber's internal surface. In the chamber which was relatively low, ethyl alcohol was applied for fixing the invaded cell. Next, 0.1% crystal violet was applied to stain the collect cells for 15 min. Cell number was manually counted.

### 2.9. Subcellular Fractionation

Cytoplasmic and nuclear separations were carried out with the use of PARIS Kit (Life Technologies, Hangzhou, Zhejiang, China) based on the producer's guideline.

### 2.10. RNA Pull-Down Assay

Biotinylated TSPEAR-AS2 probe, miR-487a-3p probe, and the relative control were obtained according to GenePharma (Shanghai, China). Cellular lysates were combined with M-280 streptavidin magnetic beads (Invitrogen) as discussed in prior studies [18], and qRT-PCR was used to detect the TSPEAR-AS2 or miR-487a-3 expression.

### 2.11. Luciferase Reporter Assay

TSPEAR-AS2-WT/MUT or PPM1A-WT/MUT was subcloned into the pmirGLO dual-luciferase vector (Biomart, Haidian, Beijing, China). First, SCC25 or FADU cells received the cotransfection by using pmirGLO-TSPEAR-AS2-WT/MUT and NC mimics or miR-487a-3p mimics. Second, SCC25 or FADU cells received the cotransfection by using miR-487a-3p mimics and pmirGLO-PPM1A-WT/MUT. Luciferase and renilla signals received the measurement 48 h after being transfected with the use of the Dual-Luciferase Reporter Assay System (Promega, Pudong, Shanghai, China). Luciferase reporter assays were conducted in triplicate.

### 2.12. Western Blot Assay

SCC25 and FADU cells were harvested and lysed in RIPA Lysis Buffer (Yita Biology, Pinggu, Beijing, China) to collect proteins. Proteins were separated using SDS-PAGE at 30 *μ*g/lane and transferred to a nitrocellulose membrane (EMD Millipore, Pudong, Shanghai, China). Next, our group blocked the membrane with 5% dried skimmed milk for 1 h. Then, the membrane was incubated with primary antibodies including anti-PPM1A (Cat no. ab14824, Abcam) and anti-GAPHD (Cat no. ab8245; Abcam) at 4°C throughout the night and with horseradish peroxidase- (HRP-) conjugated secondary antibodies (Guduo, Shanghai, China) for 1 h under ambient temperatures. The researchers employed ImageJ software for quantifying the density exhibited by the respective band.

### 2.13. Statistical Analysis

Data had the presentation of mean ± standard deviation (SD). A statistic-based investigation was conducted with the use of SPSS (IBM, Armonk, NY, USA) and diagrams received the mapping process with the use of GraphPad Prism software. For comparison within different groups, Student's *t*-test or one-way ANOVA was performed. The OS and DFS were analyzed by log-rank test, and survival curves were plotted according to Kaplan-Meier. The Cox proportional hazards model was employed for the multivariate analysis. The statistical significance (*P* value) is set as less than 0.05.

## 3. Results

### 3.1. Expression of TSPEAR-AS2 in OSCC Tissues and Adjacent Normal Tissues

**Figure 1:**
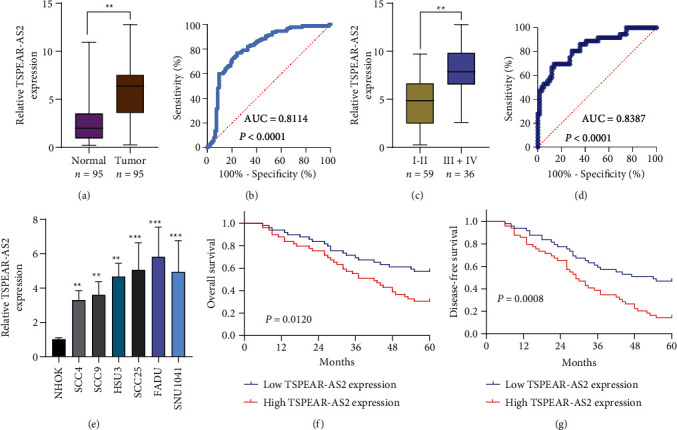
The distinct upregulation of TSPEAR-AS2 in OSCC patients and its clinical significance. (a) TSPEAR-AS2 level was distinctly greater within OSCC tissue as compared with that in normal tissues. (b) ROC curve analysis for the detection of CRC using TSPEAR-AS2. (c) The comparison of TSPEAR-AS2 levels in OSCC specimens with I-II or III-IV. (d) TSPEAR-AS2 can be used to distinguishing OSCC specimens with III-IV from those with I-II. (e) Relative TSPEAR-AS2 expression was measured by qRT-PCR in six OSCC cells and NHOK cells. (f and g) The correlation of TSPEAR-AS2 expression with OS (f) and DFS (g) of OSCC patients analyzed by Kaplan-Meier analysis. ^∗∗∗^*P* < 0.001, ^∗∗^*P* < 0.01.

For exploring the potential role of TSPEAR-AS2 in OSCC, the expression of TSPEAR-AS2 was analyzed in OSCC tissues and cell lines. As shown in Figure 1(a), compared with normal group, the expression of TSPEAR-AS2 was distinctly increased in OSCC tissues (*P* < 0.05). ROC assays showed strong separation between the two groups (tumor group vs. normal group), with an AUC of 0.8114 (0.7478-0.8749) (Figure 1(b)). In addition, higher levels of TSPEAR-AS2 were observed in OSCC specimens with III-IV compared with those with I-II (Figure 1(c)). ROC assays showed strong separation between the two groups (I-II vs. III-IV), with an AUC of 0.8387 (0.7550-0.9225) (Figure 1(d)). Furthermore, our group also observed that TSPEAR-AS2 expression was distinctly upregulated in six OSCC cells compared with NHOK cells (Figure 1(e)).

### 3.2. The Prognostic Value of TSPEAR-AS2 Expression in OSCC Patients

To better understand the potential roles of TSPEAR-AS2 in OSCC development, the patients were divided into high and low expression groups by the median expression level of TSPEAR-AS2 (5.89). The Chi-square test revealed that high TSPEAR-AS2 expression was associated with the advanced TNM stage (*P* = 0.022) (Table 2). However, there was no association between TSPEAR-AS2 expression and other clinical factors (all *P* > 0.05). With five-year follow-up by the apartment of our hospital, we collected five-year survival data, which was analyzed by Kaplan-Meier analysis and log-rank test. We found that the patients in the high TSPEAR-AS2 expression group had shorter overall survival (OS, *P* = 0.0120, Figure 1(f)) and disease-free survival (DFS, *P* = 0.0008, Figure 1(g)) than those in the low TSPEAR-AS2 expression group. More importantly, after multivariate analyses of prognostic factors in OSCC patients, the TSPEAR-AS2 expression level was identified to be an independent prognostic factor for OS (HR = 2.893, 95% CI: 1.217-4.324; *P* = 0.014), as well as DFS (HR = 3.015, 95% CI: 1.334-4.732; *P* = 0.007) of OSCC patients (Table 3).

### 3.3. The Effects of TSPEAR-AS2 Knockdown in OSCC Progression

**Figure 2:**
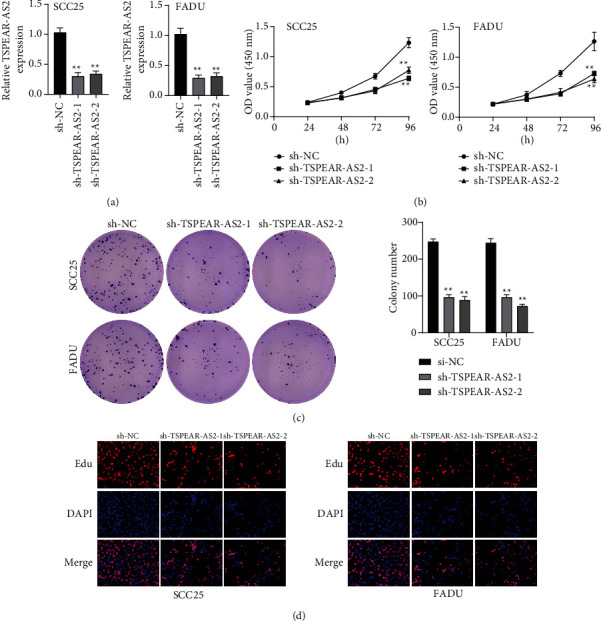
TSPEAR-AS2 depletion inhibits OSCC proliferation. (a) Relative expression of TSPEAR-AS2 after transfection with shRNAs. (b) CCK8 assay for cell proliferation analysis. (c) Colony formation test was carried out for cell proliferation after transfection of sh-TSPEAR-AS2-1 or sh-TSPEAR-AS2-2. (d) TSPEAR-AS2 knockdown inhibited cell proliferation, as determined by EdU assays. ^∗∗^*P* < 0.01.

**Figure 3:**
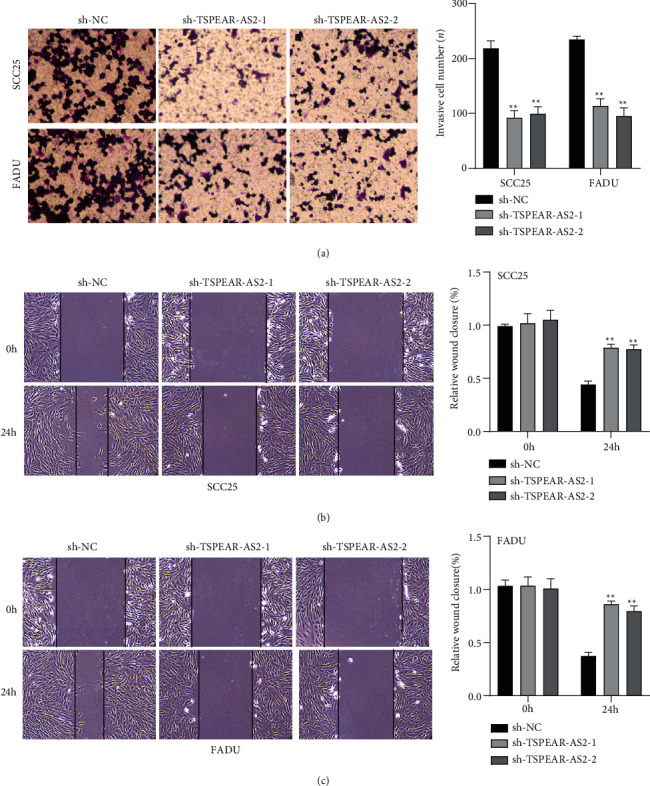
Knockdown of TSPEAR-AS2 suppressed the migrating and invading processes of OSCC cell. (a) Cell migration ability was detected in SCC25 and FADU cells in which TSPEAR-AS2 expression was decreased. (b, c) Transwell assay showed that knockdown of TSPEAR-AS2 could regulate SCC25 and FADU cells' invasive. ^∗∗^*P* < 0.01.

For exploring the regulating effect exerted by TSPEAR-AS2 in OSCC cells, three shRNAs (sh-TSPEAR-AS2-1 and sh-TSPEAR-AS2-2) targeted to TSPEAR-AS2 and one scrambled control shRNA (sh-NC) were applied. The efficiency was then determined in stably transfected cells by RT-qPCR (Figure 2(a)). CCK-8 results showed that the proliferation of TSPEAR-AS2 knockdown cells received the significant inhibition in contrast to the negative control and the blank group in both SCC25 and FADU cell lines (Figure 2(b)). Colony formation assay and Edu assays also showed that knockdown of TSPEAR-AS2 considerably suppressed the viability of SCC25 and FADU cells (Figures 2(c) and 2(d)). Subsequently, we performed wound healing assay and transwell assay to explore the effects of TSPEAR-AS2 knockdown on metastatic abilities of OSCC cells. It was observed that knockdown of TSPEAR-AS2 visibly reduced the migrative (Figure 3(a)) and invasive (Figures 3(b) and 3(c)) abilities of SCC25 and FADU.

### 3.4. TSPEAR-AS2 Served as a Sponge of miR-487a-3p

**Figure 4:**
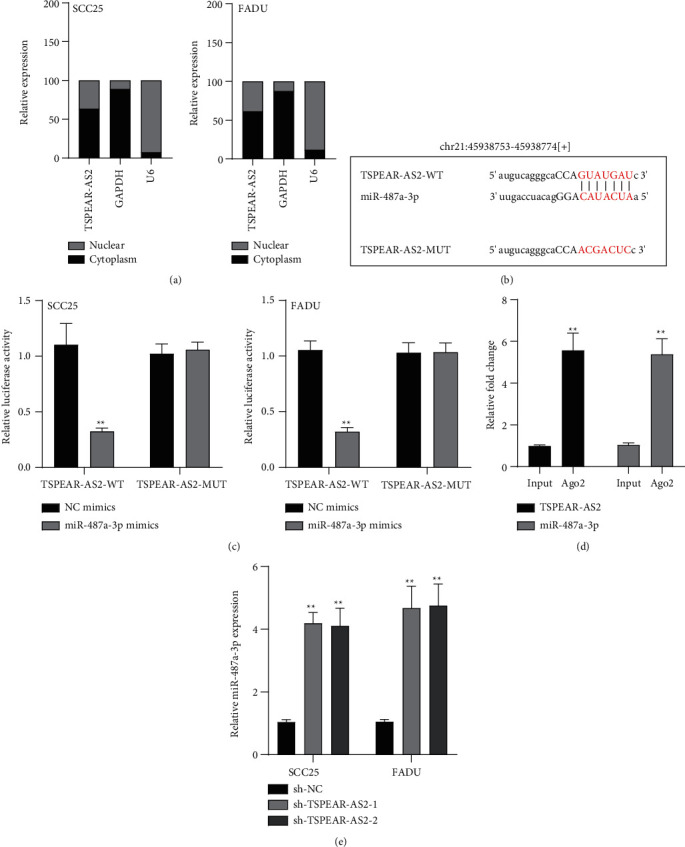
TSPEAR-AS2 acts as a molecular sponge of miR-487a-3p. (a) Relative TSPEAR-AS2 expression levels in nuclear and cytosolic fractions of SCC25 and FADU cells. (b) The binding sites of TSPEAR-AS2 with miR-487a-3p by Starbase 2.0. (c) According to luciferase reporter tests, miR-487a-3p reduced the luciferase activity of TSPEAR-AS2-WT, other than of TSPEAR-AS2-MUT. (d) RNA pull-down assays. (e) RT-PCR for miR-487a-3p expressing state in SCC25 and FADU cells after TSPEAR-AS2 knockdown. ^∗∗^*P* < 0.01.

It has been demonstrated that numerous cytoplasmic lncRNAs have been reported to be competing endogenous RNAs (ceRNAs) through the competitive bind process of microRNAs [19, 20]. With the use of the subcellular fractionating process, TSPEAR-AS2 had the expression within the nucleus and cytoplasm, and a greater proportion of TSPEAR-AS2 was observed in the cytoplasm (Figure 4(a)). Bioinformatics tools estimated the complementary binding site in TSPEAR-AS2 and miR-487a-3p, which was confirmed using the luciferase reporter assay (Figures 4(b) and 4(c)). RNA pull-down assays also demonstrated the combination between TSPEAR-AS2 and miR-487a-3p (Figure 4(d)). Finally, RT-PCR assays revealed that after TSPEAR-AS2 expression was suppressed, the levels of miR-487a-3p were increased in SCC25 and FADU (Figure 4(e)).

### 3.5. PPM1A Was Identified as a Direct Target of miR-487a-3p in OSCC Cells

**Figure 5:**
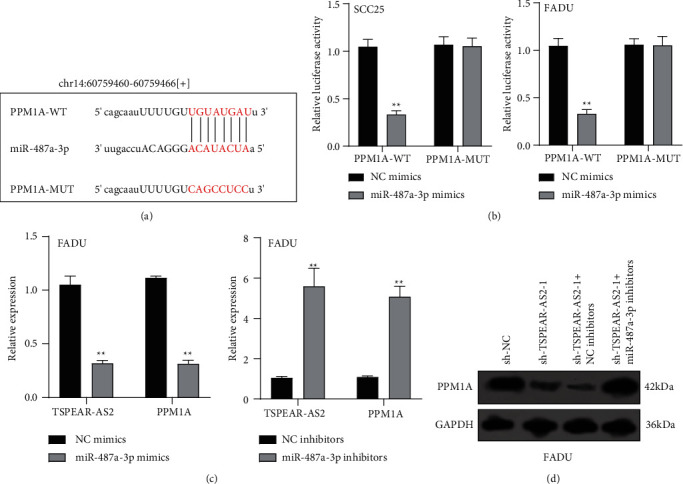
PPM1A was miR-487a-3p direct target gene. (a) Direct binding sites in miR-487a-3p and PPM1A were presented. (b) Luciferase reporter assay validated the molecular binding. (c) RT-PCR for the expressing states of miR-487a-3p and TSPEAR-AS2 in FADU cells after miR-487a-3p overexpression or knockdown. (d) Western blot assays determined the expression of PPM1A in FADU cells under the transfection by using sh-NC, sh-TSPEAR-AS2-1, sh-TSPEAR-AS2-1+ NC inhibitors, or sh-TSPEAR-AS2-1+ miR-487a-3p inhibitors. ^∗∗^*P* < 0.01.

To explore the specific mechanism of the TSPEAR-AS2/miR-487a-3p axis, we predicted the potential targets of miR-487a-3p by using TargetScan. The results showed that PPM1A might be a potential target of miR-487a-3p (Figure 5(a)), which was demonstrated with the use of luciferase reporter assays (Figure 5(b)). In addition, we observed that miR-487a-3p overexpression distinctly suppressed the levels of TSPEAR-AS2 and PPM1A, while miR-487a-3p knockdown displayed an opposite effect (Figure 5(c)). Further rescue experiments revealed that knockdown of miR-487a-3p distinctly reversed the suppression of TSPEAR-AS2 knockdown on the expression of PPM1A (Figure 5(d)) in FADU cells.

## 4. Discussion

The identification of sensitive biomarkers was very important for the improvements of the clinical outcome of OSCC patients [21, 22]. There have been many papers reporting the discovery of OSCC biomarkers, but only a few biomarkers have been validated and successfully applied in routine clinical practice [23, 24]. Moreover, most biomarkers possess limitations for the early detection of OSCC, and their prognostic value was plagued by inaccuracies. In recent years, more and more studies highlighted the potential of lncRNAs used as novel biomarkers for cancer patients [25, 26]. Several lncRNAs, such as lncRNA HOXA11-AS, lncRNA CASC9, and lncRNA LEF1-AS1, have been shown to possess diagnostic and prognostic values for OSCC patients [27–29]. In this study, we identified a novel OSCC-related lncRNA, TSPEAR-AS2 which was highly expressed in OSCC and could be used as a diagnostic and prognostic marker for OSCC patients. The OS and DFS of OSCC patients with high TSPEAR-AS2 expression were distinctly shorter than those with low TSPEAR-AS2 expression, which was consistent with the prognostic value of TSPEAR-AS2 expression in gastric cancer patients [17]. However, the sample size was relatively small. We will collect more samples for research in the future.

Because of the role of cell signaling pathways in cancer initiation, progression, and metastasis, lncRNAs involved in these pathways can influence all aspects of tumorigenesis [30, 31]. Therefore, lncRNAs may play a role in carcinogenesis or tumor inhibition. For instance, suppression of TTN-AS1 resulted in an ability inhibition of OSCC cells in the tumor growth and metastasis via miR-411-3p/NFAT5 axis [32]. Overexpression of MCM3AP-AS1 promoted the proliferation and invasion of OSCC cells via regulating miR-204-5p/FOXC1 [33]. Given that TSPEAR-AS2 was highly expressed in OSCC specimens and predicted a poor prognosis of OSCC patients, we performed loss-of-function assays, finding that knockdown of TSPEAR-AS2 distinctly suppressed the proliferation, migration, and invasion of OSCC cells. In the future, in vivo assays were needed to further demonstrate the effects of TSPEAR-AS2 on OSCC progression. Previously, the similar oncogenic roles of TSPEAR-AS2 on gastric cancer cells were also demonstrated, suggesting the great potential of TSPEAR-AS2 used as a novel therapeutic target [17].

As revealed in existing research, miRNA is carcinogenic or inhibitory within tumorigenesis, and the expressions achieved by lncRNAs are able to control the activities of miRNAs [34, 35]. Increasing evidences show that lncRNAs control OSCC to develop and progress by sponging an array of downstream miRNAs [36, 37]. Thus, delving into the mentioned miRNAs can help develop feasible approaches for preventing and treating the OSCC. Our experiments demonstrated TSPEAR-AS2 had a major expression within the cytoplasm, suggesting the tremendous possibility of TSPEAR-AS2 acting as a ceRNA. Starbase 2.0 revealed miR-487a-3p may be a target of TSPEAR-AS2, which was further confirmed by Luciferase Reporter Gene Assay, RNA pull-down, and RT-PCR. Previously, miR-487a-3p has been reported to be overexpressed in several tumors, such as colon cancer and gastric cancer [38, 39]. Importantly, in OSCC, miR-487a-3p was found to display an upregulated level and suppress the proliferation and metastasis of OSCC cells [40]. These findings suggested TSPEAR-AS2 may display its oncogenic roles via sponging miR-487a-3p.

PPM1A refers to a protein phosphatase 2C family member of Ser/Thr protein phosphatases [41]. It can control TGF-beta/Smad19-21 and mitogen-activated protein kinase22 cellular signaling channels, and proliferating, invading, and migrating processes of cells [42, 43]. In OSCC, PPM1A has been reported to be highly expressed and promoted the proliferation and metastasis of OSCC cells, whereas how PPM1A controls the mentioned activities requires in-depth studies [40]. In this study, we found PPM1A may be a target of miR-487a-3p. Overexpressed miR-487a-3p suppressed the expression of PPM1A, while miR-487a-3p knockdown displayed an opposite effect. Based on the oncogenic roles of PPM1A in OSCC progression, we supposed that miR-487a-3p may serve as a tumor suppressor via targeting PPM1A. Finally, we performed rescue experiments, finding that knockdown of miR-487a-3p distinctly reversed the suppression of TSPEAR-AS2 knockdown on the expression of PPM1A protein. Thus, our findings suggested that TSPEAR-AS2 may display its suppression on the abilities of the proliferating, migrating, and invading processes pertaining to OSCC cell by increasing PPM1A expressing level by sponging miR-487a-3p.

## 5. Conclusion

To sum up, we identified TSPEAR-AS2 as a tumor-driver within OSCC, and the greater expressing state of TSPEAR-AS2 showed a relationship to tumor metastasis and poor prognosis. TSPEAR-AS2/miR-487a-3p/PPM1A axis may act as a new ceRNA regulatory network, thus, accelerating the malignant processes of OSCC. TSPEAR-AS2 may become a novel biomarker and therapeutically related target for this disease in the future.

## Figures and Tables

**Table 1:** The primers used in this study for RT-PCR.

Names	Sequences (5′-3′)
TSPEAR-AS2: F	ACCCTCGACGTCCGTCCACGG
TSPEAR-AS2: R	GCAGGCCATGCAAGTCACAG
miR-487a-3p: F	ATGGCGGAATCATACAGGGAC
miR-487a-3p: R	CTCAACTGGTGTCGTGGAGTC
PPM1A: F	AGGGGCAGGGTAATGGGTT
PPM1A: R	GATCACAGCCGTATGTGCATC
GAPDH: F	GGAGCGAGATCCCTCCAAAAT
GAPDH: R	GGCTGTTGTCATACTTCTCATGG
U6: F	GCGCGTCGTGAAGCGTTC
U6: R	GTGCAGGGTCCGAGGT

**Table 2:** Relationship between lncRNA TSPEAR-AS2 expression and clinicopathological characteristics.

Variable	Cases (*n*)	TSPEAR-AS2 expression		*P* values
		High	Low	
Age				0.745
<60	45	24	21	
≥60	50	25	25	
Gender				0.972
Male	58	30	28	
Female	37	19	18	
Histology/differentiation				0.082
Well + moderate	57	26	31	
Poor	38	23	15	
TNM stage				0.022
I + II	59	25	34	
III + IV	36	24	12	

**Table 3:** Multivariate analyses of prognostic factors in OSCC patients.

Variables	Overall survival			Disease-free survival		
	HR	95% CI	*P* value	HR	95% CI	*P* value
Age	0.783	0.453-1.343	0.459	0.821	0.445-1.532	0.321
Gender	0.556	0.341-1.231	0.244	0.671	0.445-1.435	0.329
Histology/differentiation	1.132	0.673-1.873	0.112	1.345	0.792-1.832	0.093
TNM stage	3.132	1.325-4.789	0.005	3.436	1.429-5.554	0.001
TSPEAR-AS2 expression	2.893	1.217-4.324	0.014	3.015	1.334-4.732	0.007

## Data Availability

The data used to support the findings of this study are available from the corresponding author upon request.
